# Does calibration technique for distal locking screw insertion reduce radiation exposure and operative time during intramedullary nailing of humeral shaft fractures in comparison with freehand technique?

**DOI:** 10.1016/j.sipas.2025.100307

**Published:** 2025-09-08

**Authors:** Mirza Sivro, Tarik Branković

**Affiliations:** Department of Orthopedics and Traumatology, Cantonal Hospital Zenica, Zenica, Bosnia and Herzegovina

**Keywords:** Bone screws, Humeral fractures, Intramedullary nail, Radiation exposure

## Abstract

**Purpose:**

The aim of this study was to compare the influence of two different methods of distal screw insertion during intramedullary nailing of humeral shaft fractures on radiation exposure and operative time.

**Methods:**

A single-center retrospective study, which included 44 patients, was conducted. Patients were divided into the Freehand and Calibration groups according to the technique used for distal screw insertion. Medical records were used to collect baseline characteristics of patients and complications, and operative reports were used to collect data for outcomes which included number of expositions, dose area product (DAP), fluoroscopy time and operation time. Operation time was defined as time from skin incision to final suture.

**Results:**

There were no significant differences noted between the groups in gender, age, fracture side and fracture type distribution. The mean DAP in the Calibration group measured 232.8 ± 130.1 μGy·m2, and was lower than in the Freehand group where measured value was 305.4 ± 141.6 μGy·m2, without significant difference between the groups (*p* = 0.084). Mean fluoroscopy time was also lower in the Calibration group of patients (32.3 ± 12.7 s) than in the Freehand group (39.4 ± 14.6 s), with *p* = 0.094. Mean operative time was shorter in the Calibration group (68.8 ± 27.1 min) in comparison with the Freehand group (76.5 ± 17.7 min), but without statistically significant difference (*p* = 0.272).

**Conclusion:**

The usage of calibration technique for distal locking screw insertion has similar operative time and intraoperative radiation exposure during intramedullary nailing of humeral shaft fractures compared with the freehand technique.

## Highlights

• Intramedullary nailing of humeral shaft fractures is associated with radiation exposure.
• Various techniques for aiming distal locking screws are used.
• Freehand technique is being the most common one.
• Calibration technique may suggest a tendency towards reduction of radiation exposure.

## Introduction

Humeral shaft fractures occur equally in males and females across all age groups with incidence of 1 % to 2 % of all fractures [1]. They are mostly caused by low-energy trauma in older patients, and by high-energy trauma in younger ones [2]. Intramedullary nailing of humeral shaft fractures is associated with lower intraoperative blood loss, shorter hospitalization and operative time, early weight bearing of the extremity and high rates of union [3]. Antegrade and retrograde locked humeral nails have become standard treatment for diaphyseal humeral fractures with mechanical and biological advantages which include load-sharing properties and avoidance of fracture site exposure [4,5]. However, the disadvantage of using humeral nailing techniques is radiation exposure. Ionizing radiation has two types of deleterious radiation effects: non stochastic and stochastic [6]. Recommendations and guidelines from International Commission on Radiological Protection (IRCP) are used and implemented in order to reduce hazards of radiation exposure [7]. Insertion of distal locking screws during nailing of fractures of long bones can be a demanding and time-consuming process which takes up to one-third-part of the surgery. Various methods, which rely mainly on fluoroscopy, have been proposed for distal aiming such as nail mounted targeting devices, freehand technique, and electromagnetic-guided systems [8]. Electromagnetic-guided systems for distal aiming are expensive and unavailable in the vast majority of hospitals [9]. Before the year 2022 at our institution, long intramedullary nail (Long Diphos Nail®, Lima Corporate, Italy) was used for nailing of humeral shaft fractures where freehand technique was applied for aiming of distal screws. At the beginning of the year 2022 our institution switched to a new intramedullary nail (Thales Humeral Nail®, 7 s Medical, Switzerland). This humeral nailing system uses a new calibration technique for distal aiming in order to avoid failure of distal screw insertion through the hole due to nail twist and deformation as they are passed into the intramedullary canal. Novelty of our research is an investigation of a role of nail mounted targeting device in reduction of intraoperative radiation exposure and intraoperative time that has not been yet reported in the literature.

The aim of this study was to compare the influence of two different methods of distal screw insertion during intramedullary nailing of humeral shaft fractures on radiation exposure and operative time.

## Patients and study design

A single-center retrospective study, which included 44 patients, was conducted. Inclusion criteria were: humeral shaft fractures according to the AO/OTA classification types 12. A, B and C [10], closed fractures, patients who signed the informed consent, bipolar interlocking (two screws proximal and one distal), and patients older than 18 years. Exclusion criteria were: fractures older than 2 weeks, pathological fractures, associated fractures, polytraumatized patients, open fractures, fractures that required open reduction, failure of calibration technique. This study was performed in line with the principles of the Declaration of Helsinki. Approval was granted by the Ethics Committee of the Cantonal Hospital Zenica (Date: 30.06.2024. / No. 00–03–35–968–18/25). Informed consent was obtained from all individual participants included in the study.

## Methods

Between January 2019 and December 2021, 22 patients who were treated with long intramedullary nail (Long Diphos Nail®, Lima Corporate, Italy) met inclusion criteria and were marked as Freehand group. Between January 2022 and December 2024, 22 patients treated with Thales Humeral Nail® (7 s Medical, Switzerland) met the inclusion criteria and were marked as a Calibration group. Medical records were used to collect baseline characteristics of patients and complications, and operative reports were used to collect data outcomes which included number of expositions, dose area product (DAP), fluoroscopy time and operation time. Dose area product (DAP) meters, integrated in the C-arm device, measure radiation dose emitted from the X-ray tube, multiplied by the area of the X-ray field. Operation time was defined as time from skin incision to final suture. All operations were performed by four surgeons with a high level of expertise who were familiar with both techniques.

### Operative technique

Operative technique was the same for both groups up to distal screw insertion. Length and diameter of the nails were determined preoperatively. Patients were placed in a beach-chair position. Longitudinal incision was made slightly anterolateral to the acromion, deltoid and supraspinatus muscle were split, and under the image intensification entry point was made in the medial tip of the tuberculum majus. A medullary canal was opened using the awl and guide wire was introduced into the canal up to the fracture site. Closed reduction of the fracture was made and wire was advanced under image intensification up to the olecranon fossa. The canal was gradually enlarged with a flexible reamer to a size 0,5 cm greater than the nail diameter. The appropriate nail was connected to the insertion handle and inserted into the canal and advanced to the appropriate length across the fracture site under image intensification, and was sunken 5 mm under the humeral bone surface. Two proximal locking screws were inserted using an assembled aiming device.

### Freehand technique for distal locking

In the Freehand group, one distal screw was inserted from lateral to medial using freehand technique. The image intensifier was first used to localize a perfectly round circle of the interlocking screw hole lined up in the intensifier beam. A small longitudinal skin incision was made and a sharp drill bit was angulated obliquely to allow imaging, and the tip was placed in the exact center of the projected hole as confirmed by the lateral fluoroscopic image. The drill was then positioned parallel to the path of the beam and advanced. The drill was then removed, leaving the drill bit in position to confirm the direction and placement. The drill was then advanced through the nail, a depth gauge was used to determine the screw length, and a locking screw was inserted.

### Calibration technique for distal locking

**Fig. 1:**
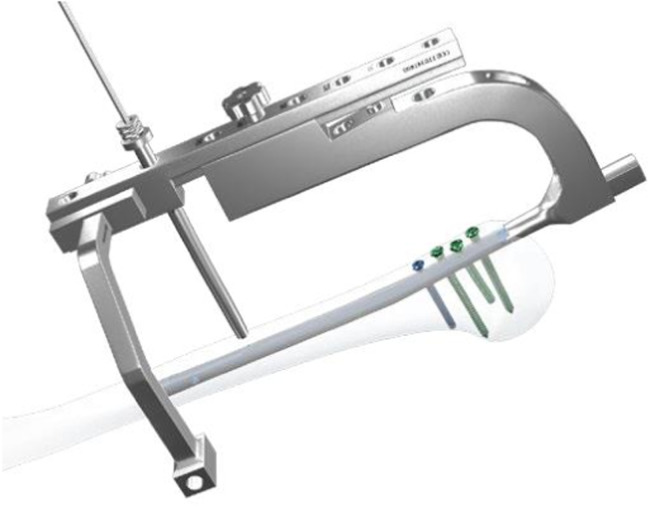
Installing aiming arms and drilling of the near cortex.

**Fig. 2:**
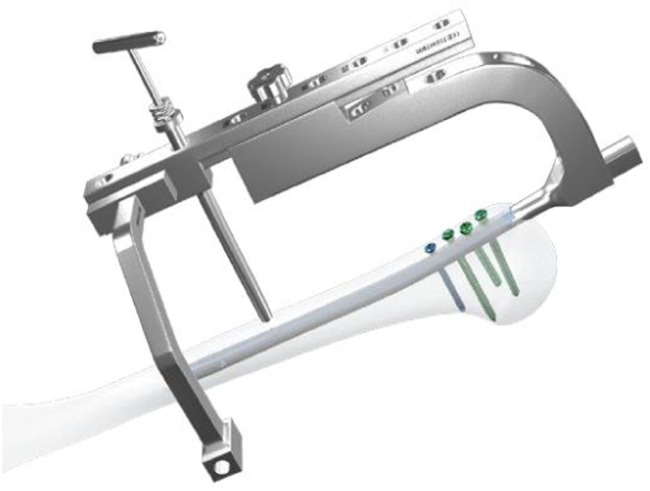
Clearing the debris.

**Fig. 3:**
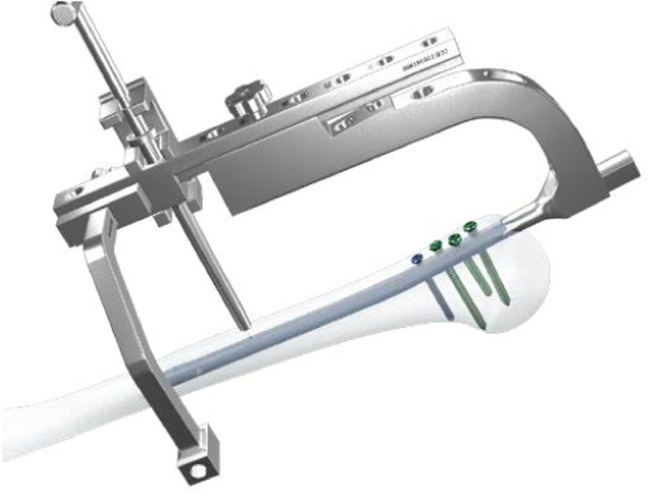
Assembling the calibrating pin.

**Fig. 4:**
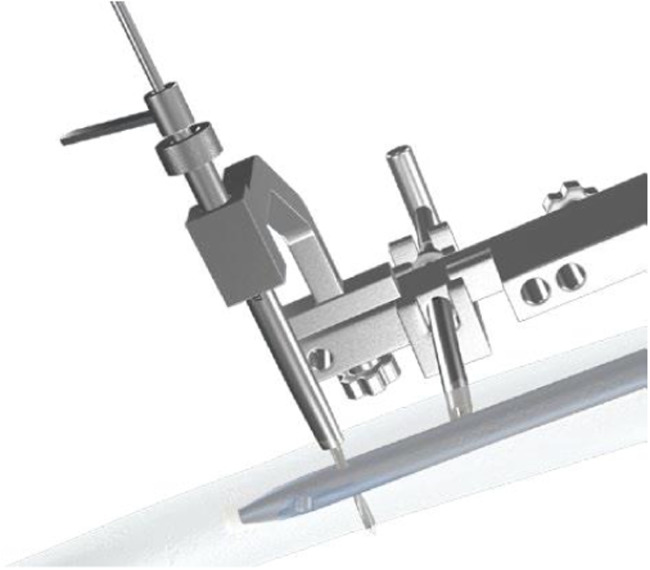
Drilling for distal locking.

In the Calibration group distal screw was also inserted from the lateral to medial with calibration technique. After removing the proximal aiming device, an aiming arm for distal screw insertion was installed. Stab incision was made and trocar inserted to the bone (Fig. 1). A 3.0 mm drill bit was advanced through the drill sleeve and near cortex was drilled. The drill bit was removed and a 3.2 mm drill bit with a flat head was inserted to the nail to remove the debris (Fig. 2). The drill bit and drill sleeve were removed, and a calibrating pin was placed ensuring its contact with the nail platform. The U-Clip was then used to connect the calibrating pin and the straight aiming arm (Fig. 3). A protection sleeve and trocar were inserted through the curved aiming arm to the bone. Trocar was then removed and the inner sleeve was inserted through which drill bit was advanced through both cortices after which depth gauge was inserted and screw with determined length inserted from lateral to medial (Fig. 4). Screw position was checked under the image intensification.

In all patients cefazolin was used for antibiotic prophylaxis and dalteparin was used for thromboprophylaxis.

### Statistical analysis

Estimated sample size was 44 participants, when an independent 2-sample *t*-test with 80 % power at the 0.05 level of significance was performed based on a 0.60 effect size. Baseline characteristics of patients were evaluated using descriptive statistics. Shapiro-Wilk test was used to test the normality of data distribution. Since all continuous variables showed normal distribution, they were presented as mean and standard deviation, and a two-sided Student *t-* test was used to compare the means. The χ2 test or Fisher's exact test were used to analyse categorical variables. Results were considered significant with *p* < 0.05. Data analysis was performed using SPSS Statistics, Version 20 (SPSS, Inc., Chicago, IL, USA).

## Results

There were in total 44 patients included in the study who were divided, based on the distal screw insertion technique, into the Freehand and Calibration group, each consisting of 22 patients. There was no significant difference in gender distribution across groups (*p* = 0.760). Patients in the Calibration group were slightly younger (49.7 ± 19.8 years) than patients in the Freehand group (51.5 ± 14.9 years), but without statistically significant difference (0.745). No statistical differences were noted between the groups regarding body mass index (BMI) (*p* = 0.280). There were no significant differences in fracture side (*p* = 1.000) and fracture type distribution (*p* = 0.437) between the groups (Table 1). There was one case of postoperative radial nerve palsy in the Freehand group. The mean number of expositions made with the C-arm was 127.6 ± 47 in the Calibration group, and was lower than the number of expositions in the Freehand group (151.7 ± 55.8), without significant difference (Table 2). The mean DAP in the Calibration group measured 232.8 ± 130.1 μGy·m2, and was lower than in the Freehand group where measured value was 305.4 ± 141.6 μGy·m2, without significant difference between the groups (*p* = 0.084). Mean fluoroscopy time was also lower in the Calibration group of patients (32.3 ± 12.7 s) than in the Freehand group (39.4 ± 14.6 s), with *p* = 0.094. Mean operative time was shorter in the Calibration group (68.8 ± 27.1 min) in comparison with the Freehand group (76.5 ± 17.7 min), but without statistically significant difference (*p* = 0.272) (Table 2).

**Table 1:** Baseline characteristics of patients and complications.

Variables	Freehand group (*N* = 22)	Calibration group (*N* = 22)	p value
**Gender (No,%)**			
Males	14 (31.8 %)	12 (27.3 %)	0.760
Females	8 (18.2 %)	10 (22.7 %)	
**Age (mean ± SD)**	51.5 ± 14.9	49.7 ± 19.8	0.745
**BMI (mean ± SD)**	23.83 ± 4.48	24.52 ± 5.78	0.280
**Fracture side (No,%)**			
Right	11 (25 %)	11 (25 %)	1.000
Left	11 (25 %)	11 (25 %)	
**Fracture type (AO/OTA) (No,%)**			
A1	5 (11.4 %)	4 (9.1 %)	0.437
A2	4 (9.1 %)	6 (13.6 %)	
A3	6 (13.6 %)	7 (15.9 %)	
B1	2 (4.5 %)	0 (0.0 %)	
B2	2 (4,5 %)	1 (2.3 %)	
B3	0 (0.0 %)	1 (2.3 %)	
C1	1 (2.3 %)	3 (6.8 %)	
C2	2 (4.5 %)	0 (0.0 %)	
C3	0 (0.0 %)	0 (0.0 %)	
**Complications**			
Radial nerve palsy	1	0	0.500

BMI, body mass index; AO/OTA, Arbeitsgemeinschaft für Osteosynthesefragen/Orthopedic Trauma Association classification.

**Table 2:** Differences in outcomes between the groups.

Outcome	Freehand group	Calibration group	p value
Expositions (No)	151.7 ± 55.8	127.6 ± 47	0.129
DAP (μGy·m2)	305.4 ± 141.6	232.8 ± 130.1	0.084
Fluoroscopy time (seconds)	39.4 ± 14.6	32.3 ± 12.7	0.094
Operative time (minutes)	76.5 ± 17.7	68.8 ± 27.1	0.272

DAP, dose area product.

## Discussion

Distal locking in humeral shaft fractures can be challenging due to technical demands and proximity of neurovascular structures. In the retrospective study of Hichao F et al. [11], with similar methods to our study, significant reduction of surgical time was noted in the group of patients where electromagnetic targeting system was used in comparison with the freehand technique during intramedullary nailing of humeral shaft fractures. Demographic characteristics of our patients were similar to those reported in this study, but we did not find a significant difference in surgical time. Also this study has not evaluated differences in radiation exposure.

Another study evaluated freehand and electromagnetic targeting system for distal screw insertion in humeral nailing. In this study, Persiani P et al. [12] found reduced radiation exposure during insertion of distal screws and time needed to position the distal screw. However, they measured time for insertion of single screws and radiation exposure during their insertion, unlike in our study, where we evaluated the effects of targeting methods to the overall operative time and radiation exposure.

Barış A et al. compared freehand technique with an internal locking system in their study where they used nail from the same manufacturer for free hand technique ( Thales Humeral Nail® ) in contrast to our study where we applied calibration technique on the same implant [13]. They found freehand technique for distal locking screw insertion in humeral shaft fractures inferior to the internal locking system with significantly decreased surgical time and radiation exposure in the internal locking system group.

The study of Qi et al. [14] evaluated advantages of the robot assisted navigation system in comparison to the traditional intramedullary nailing. They found significantly shorter surgical times and fewer intraoperative fluoroscopy times in the robotic group.

Although there was no significant difference in the operative and fluoroscopy time, as well as in number of expositions and DAP values between the groups in our study, there was tendency towards lower DAP values and fluoroscopy time in the Calibration group (*p* = 0.084 and 0.094, respectively), which is important radiation exposure reduction. Lack of significant reduction in operative time may be due to various factors, but time for assembling the aiming arms for distal locking could influence the results.

One patient in the Freehand group had postoperative radial nerve palsy. It has been reported that distal locking technique affects the rate of iatrogenic radial nerve palsy [15], especially in the lateral to medial side insertion, but patient with the complication had Holstein-Lewis type of fracture which is by itself predisposing factor for radial nerve injury. Since there was no radial nerve palsy prior to the operation, injury to the radial nerve most possibly occurred by closed manipulation and traction.

The limitation of our study is its retrospective nature. A larger number of patients is needed to further evaluate the significance of the results. Also we did not record surgical and radiation time separately for distal screw insertion, and we did not evaluate the accuracy of the calibration technique for distal screw insertion.

## Conclusion

In conclusion, our results suggest that the usage of calibration technique for distal locking screw insertion, as a part of nail mounted targeting device, has similar operative time and intraoperative radiation exposure during intramedullary nailing of humeral shaft fractures compared with the freehand technique.

## Funding

No funding was received to assist with the preparation of this manuscript.

## CRediT authorship contribution statement

**Mirza Sivro:** Writing – original draft, Validation, Resources, Formal analysis, Conceptualization. **Tarik Branković:** Writing – review & editing, Supervision, Investigation, Conceptualization.

## Declaration of competing interest

The authors declare that they have no known competing financial interests or personal relationships that could have appeared to influence the work reported in this paper.

## Appendix Supplementary materials

Image, application 1

## References

[1] Updegrove G.F., Mourad W., Abboud J.A. (2018). Humeral shaft fractures. J Shoulder Elbow Surg.

[2] Rolueau D.M., Bastard C., Branes R., Chapleau J., Tornetta P., Ricci W.M., Ostrum R.F., McKee M.D., Olliviere B.J., de Ridder V.A. (2024). Rockwood and green’s fractures in adults.

[3] Mocini F., Cazzato G., Masci G., Malerba G., Liuzza F., Maccauro G. (2020). Clinical and radiographic outcomes after antegrade intramedullary nail fixation of humeral fractures. Injury.

[4] Orapiriyakul W., Apivatthakakul V., Theppariyapol B., Apivatthakakul T. (2023). Humerus shaft fractures, approaches and management. J Clin Orthop Trauma.

[5] Obada B., Zekra M., Iliescu D.M., Popescu I.A., Costea D.O., Petcu L.C. (2022). Antegrade intramedullary locking nail in the management of proximal and middle thirds of humeral diaphyseal fractures. Int Orthop.

[6] Ott M., McAlister J., VanderKolk W.E., Goldsmith A., Mattice C., Davis A.T. (2006). Radiation exposure in trauma patients. J Trauma.

[7] Buckley R.E., Moran C.G., Apivatthakakul T. (2017).

[8] Browner B.D. (1996).

[9] Antonini G., Stuflesser W., Crippa C., Touloupakis G. (2016). A distal-lock electromagnetic targeting device for intramedullary nailing: suggestions and clinical experience. Chin J Traumatol.

[10] Müller M.E., Koch P., Nzarian S., Schatzker (1990).

[11] Huichao F., Xiaoming W. (2020). Reduced Surgical Time and Higher Accuracy of Distal Locking with the Electromagnetic Targeting System in Humeral Shaft Intramedullary Nailing. Orthop Surg.

[12] Persiani P., Gurzi M., Moreschini O., Di Giacomo G., Villani C. (2017). Fluoroscopic freehand and electromagnetic-guided targeting system for distal locking screws of humeral intramedullary nail. Musculoskelet Surg.

[13] Barış A., Öztürkmen Y. (2021). Comparison of humeral intramedullary nail internal locking system and standard external locking system. Istanbul Med J.

[14] Qi H., Ai X., Ren T., Li Z., Zhang C., Wu B. (2024). A clinical study on robot navigationassisted intramedullary nail treatment for humeral shaft fractures. BMC Musculoskelet Disord.

[15] Greiner F., Kaiser G., Kleiner A., Brugger J., Aldrian S., Windhager R. (2023). Distal locking technique affects the rate of iatrogenic radial nerve palsy in intramedullary nailing of humeral shaft fractures. Arch Orthop Trauma Surg.

