# Supplementary material for: Does calibration technique for distal locking screw insertion reduce radiation exposure and operative time during intramedullary nailing of humeral shaft fractures in comparison with freehand technique?
Source: Surg Pract Sci. 2025 Sep 8;23:100307. doi: 10.1016/j.sipas.2025.100307 (PMC12746272; doi:10.1016/j.sipas.2025.100307)
Supplement: Supplementary file 1 [file mmc1.docx]

STROBE Statement—Checklist

|  | Item No | Recommendation |  |  | Page No / Information |
| --- | --- | --- | --- | --- | --- |
| **Title and abstract** | 1 | (*a*) Indicate the study’s design with a commonly used term in the title or the abstract |  |  | Abstract (p.2): 'retrospective study' mentioned in Abstract. |
|  |  | (*b*) Provide in the abstract an informative and balanced summary of what was done and what was found |  |  | Abstract (p.2): Aim, methods, results, conclusion provided. |
| Introduction | | |  |  |  |
| Background/rationale | 2 | Explain the scientific background and rationale for the investigation being reported |  |  | Introduction (p.3): Radiation exposure, distal screw techniques, need for calibration method. |
| Objectives | 3 | State specific objectives, including any prespecified hypotheses |  |  | End of Introduction (p.3): Aim to compare calibration vs freehand on radiation and operative time. |
| Methods | | |  |  |  |
| Study design | 4 | Present key elements of study design early in the paper |  |  | Abstract & Methods (p.2 and 4): Single-center retrospective comparative study. |
| Setting | 5 | Describe the setting, locations, and relevant dates, including periods of recruitment, exposure, follow-up, and data collection |  |  | Methods—Patients and study design (p.4–5): Cantonal Hospital Zenica, Bosnia and Herzegovina; Jan 2019–Dec 2024. |
| Participants | 6 | (*a*) Give the eligibility criteria, and the sources and methods of case ascertainment and control selection. Give the rationale for the choice of cases and controls |  |  | Methods (p.4–5): Inclusion/exclusion criteria; Freehand = cases before 2022; Calibration = cases after 2022. |
|  |  | (*b*) For matched studies, give matching criteria and the number of controls per case |  |  | Not applicable (no matching performed). |
| Variables | 7 | Clearly define all outcomes, exposures, predictors, potential confounders, and effect modifiers. Give diagnostic criteria, if applicable |  |  | Methods (p.4–5): Outcomes = expositions, DAP, fluoroscopy time, operative time; exposure = surgical technique. |
| Data sources/ measurement | 8* | For each variable of interest, give sources of data and details of methods of assessment (measurement). Describe comparability of assessment methods if there is more than one group |  |  | Methods (p.4–5): Medical records and operative reports; DAP from C-arm; standard procedures described. |
| Bias | 9 | Describe any efforts to address potential sources of bias |  |  | Methods & Discussion (p.6, p.7–8): Same surgeons, standardized procedures; retrospective nature noted as limitation. |
| Study size | 10 | Explain how the study size was arrived at |  |  | Statistical analysis (p.6): Sample size estimation with power analysis for 44 participants. |
| Quantitative variables | 11 | Explain how quantitative variables were handled in the analyses. If applicable, describe which groupings were chosen and why |  |  | Statistical analysis (p.6): Normality tested; means ± SD; Student t-test, χ² test used. |
| Statistical methods | 12 | (*a*) Describe all statistical methods, including those used to control for confounding |  |  | Statistical analysis (p.6): t-tests, χ², Fisher’s exact test. |
|  |  | (*b*) Describe any methods used to examine subgroups and interactions |  |  | Not applicable—no subgroup analysis. |
|  |  | (*c*) Explain how missing data were addressed |  |  | Results (p.7): No missing data reported. |
|  |  | (*d*) If applicable, explain how matching of cases and controls was addressed |  |  | Not applicable. |
|  |  | (*e*) Describe any sensitivity analyses |  |  | Not performed. |
| Results | | |  |  |  |
| Participants | 13* | (a) Report numbers of individuals at each stage of study—eg numbers potentially eligible, examined for eligibility, confirmed eligible, included in the study, completing follow-up, and analysed |  |  | Results (p.6–7): 44 patients included (22 per group). |
|  |  | (b) Give reasons for non-participation at each stage |  |  | Not applicable—retrospective with fixed sample. |
|  |  | (c) Consider use of a flow diagram |  |  | Not provided. |
| Descriptive data | 14* | (a) Give characteristics of study participants (eg demographic, clinical, social) and information on exposures and potential confounders |  |  | Table 1 (p.11): Age, gender, BMI, fracture type, side. |
|  |  | (b) Indicate number of participants with missing data for each variable of interest |  |  | No missing data noted. |
| Outcome data | 15* | Report numbers in each exposure category, or summary measures of exposure |  |  | Table 2 (p.12): DAP, fluoroscopy time, operative time, expositions. |
| Main results | 16 | (*a*) Give unadjusted estimates and, if applicable, confounder-adjusted estimates and their precision (eg, 95% confidence interval). Make clear which confounders were adjusted for and why they were included |  |  | Results (p.12, Table 2): Means ± SD, p-values; no confounder adjustment. |
|  |  | (*b*) Report category boundaries when continuous variables were categorized |  |  | Not applicable—continuous variables reported. |
|  |  | (*c*) If relevant, consider translating estimates of relative risk into absolute risk for a meaningful time period |  |  | Not applicable. |

| Other analyses | 17 | Report other analyses done—eg analyses of subgroups and interactions, and sensitivity analyses |  | Discussion (p.7–8): Comparisons with literature, subgroup considerations. |  |
| --- | --- | --- | --- | --- | --- |
| Discussion | | |  |  |  |
| Key results | 18 | Summarise key results with reference to study objectives |  | Discussion (p.7): Calibration showed trend toward lower radiation/time but not significant. |  |
| Limitations | 19 | Discuss limitations of the study, taking into account sources of potential bias or imprecision. Discuss both direction and magnitude of any potential bias |  | Discussion (p.7): Retrospective design, small sample size, lack of accuracy data. |  |
| Interpretation | 20 | Give a cautious overall interpretation of results considering objectives, limitations, multiplicity of analyses, results from similar studies, and other relevant evidence |  | Discussion & Conclusion (p.7–8): Cautious interpretation, comparison with literature, limited significance. |  |
| Generalisability | 21 | Discuss the generalisability (external validity) of the study results |  | Discussion (p.7–8): Findings may be limited due to sample size, retrospective nature; similar to previous literature. |  |
| Other information | | |  |  |  |
| Funding | 22 | Give the source of funding and the role of the funders for the present study and, if applicable, for the original study on which the present article is based |  | Funding (p.8): 'No funding was received'. |  |

*Give information separately for cases and controls.

**Note:** An Explanation and Elaboration article discusses each checklist item and gives methodological background and published examples of transparent reporting. The STROBE checklist is best used in conjunction with this article (freely available on the Web sites of PLoS Medicine at http://www.plosmedicine.org/, Annals of Internal Medicine at http://www.annals.org/, and Epidemiology at http://www.epidem.com/). Information on the STROBE Initiative is available at http://www.strobe-statement.org.
